# Phase II study of radium-223 dichloride in Japanese patients with symptomatic castration-resistant prostate cancer

**DOI:** 10.1007/s10147-017-1176-0

**Published:** 2017-08-02

**Authors:** Nobuaki Matsubara, Satsohi Nagamori, Yoshiaki Wakumoto, Hirotsugu Uemura, Go Kimura, Akira Yokomizo, Hiroaki Kikukawa, Atsushi Mizokami, Takeo Kosaka, Naoya Masumori, Yoshihide Kawasaki, Junji Yonese, Yasutomo Nasu, Satoshi Fukasawa, Takayuki Sugiyama, Seigo Kinuya, Makoto Hosono, Iku Yamaguchi, Hirokazu Tsutsui, Hiroji Uemura

**Affiliations:** 1 Division of Breast and Medical Oncology, National Cancer Center Hospital East, 6-5-1 Kashiwanoha, Kashiwa, 277-8577 Japan; 2 Department of Urology, National Hospital Organization Hokkaido Cancer Center, 2-3-54 Kikusui 4 Jo, Shiroishi-ku, Sapporo, Japan; 3 Department of Urology, Juntendo University, 2-1-1 Hongo, Bunkyo-ku, Tokyo, Japan; 4 Department of Urology, Kindai University Faculty of Medicine, 377-2, Ohno-Higashi, Osaka-Sayama, Japan; 5 Department of Urology, Nippon Medical School, 1-1-5, Sendagi, Bunkyo-ku, Tokyo, Japan; 6 Department of Urology, Harasanshin Hospital, 1-8, Taihakumachi, Hakata-ku, Fukuoka, Japan; 7 Department of Urology, National Hospital Organization Kumamoto Medical Center, 1-5 Ninomaru, Chuo-ku, Kumamoto, Japan; 8 Department of Integrative Cancer Therapy and Urology, Kanazawa University Graduate School of Medical Science, 13-1 Takaramachi, Kanazawa, Japan; 9 Department of Urology, Keio University School of Medicine, 35 Shinanomachi, Shinjuku-ku, Tokyo, Japan; 10 Department of Urology, Sapporo Medical University School of Medicine, South 1, West 16, Chuo-ku, Sapporo, Japan; 11 Department of Urology, Tohoku University Hospital, 1-1, Seiryo-machi, Aoba-ku, Sendai, Japan; 12 Department of Urology, Cancer Institute Hospital of Japanese Foundation for Cancer Research, 3-8-31, Ariake, Koto-ku, Tokyo, Japan; 13 Department of Urology, Okayama University Graduate School of Medicine, Dentistry and Pharmaceutical Sciences, 2-5-1, Shikata, Okayama, Japan; 14 Prostate Center and Division of Urology, Chiba Cancer Center, 666-2, Nitona-cho, Chuo-ku, Chiba, Japan; 15 Department of Urology, Hamamatsu University School of Medicine, 1-20-1, Handayama, Higashi-ku, Hamamatsu, Japan; 16 The Japanese Society of Nuclear Medicine, 2-28-45, Honkomagome, Bunkyo-ku, Tokyo, Japan; 17 Clinical Statistics, Bayer Yakuhin, Ltd, 2-4-9, Umeda, Kita-ku, Osaka, Japan; 18 Clinical Development Specialty Medicine, Bayer Yakuhin, Ltd, 2-4-9, Umeda, Kita-ku, Osaka, Japan; 19 Department of Urology, Yokohama City University Medical Center, 4-57, Urafune-cho, Minami-ku, Yokohama, Japan

**Keywords:** Alkaline phosphatase, Bone metastasis, Castration-resistant prostate cancer, Prostate-specific antigen, Radium-223 dichloride

## Abstract

**Background:**

Radium-223 dichloride (radium-223) is the first targeted alpha therapy approved for the treatment of castration-resistant prostate cancer (CRPC) with bone metastases. This study investigated the efficacy and safety of radium-223 in Japanese patients with symptomatic CRPC and bone metastases.

**Methods:**

In this open-label, multicenter, phase II study, patients with progressive, symptomatic CRPC and bone metastases were treated with radium-223 (55 kBq/kg, intravenously) in a 4-week cycle for six cycles. The primary endpoint was the percent change in total alkaline phosphatase (ALP) from baseline at 12 weeks. Secondary endpoints included the percent ALP change from baseline to end of treatment (EOT), ALP response rates, percent change in prostate-specific antigen (PSA) from baseline to 12 weeks and EOT, PSA response rates, overall survival (OS), and time to symptomatic skeletal events (SSEs). Adverse events were monitored throughout the study period.

**Results:**

Of the 49 Japanese patients (median age 74 years), 28 completed all infusions. Mean percent change in total ALP and PSA from baseline to 12 weeks was −19.3 and +97.4%, respectively. One-year OS and SSE-free rate at the end of active follow-up were 78 and 89%, respectively. The ALP response rate was 31%, while the PSA response rate was 6%. Grade 3/4 treatment-emergent adverse events observed in ≥10% of patients included decreased lymphocyte count (14%), anemia (14%), anorexia (10%), and bone pain (10%).

**Conclusions:**

Radium-223 is effective and well tolerated in Japanese patients with CRPC and bone metastases. Results were comparable with the Alpharadin in Symptomatic Prostate Cancer Patients (ALSYMPCA) trial.

**Clinical trial registration:**

ClinicalTrials.gov NCT01929655.

**Electronic supplementary material:**

The online version of this article (doi:10.1007/s10147-017-1176-0) contains supplementary material, which is available to authorized users.

## Introduction

Bone is the major metastatic site in prostate cancer and autopsies have revealed that 90% of patients with prostate cancer and hematogenous metastases had bone metastases [1]. Bone metastases are associated with increased skeletal-related events (SREs), which reduce quality of life, and shorter survival for patients with metastatic castration-resistant prostate cancer (mCRPC) [2, 3]. The bone environment may provide disseminated prostate cancer cells a niche for survival and proliferation, thereby increasing malignancy. Prostate cancer cells that metastasize to bone promote bone turnover, which in turn stimulates prostate cancer cells [4, 5].

Approved bone-modifying agents such as bisphosphonates and denosumab, or strontium-89, a bone-targeting beta emitter, delay the onset of SREs and provide relief from pain, but their effect on survival of CRPC patients has not been shown [6, 7].

Radium-223 dichloride (radium-223) is the first targeted alpha therapy to show a survival benefit in patients with CRPC and bone metastasis [8, 9].

Clinical studies in Caucasian patients, including Alpharadin in Symptomatic Prostate Cancer Patients (ALSYMPCA), a randomized, double-blind, placebo-controlled, pivotal phase III trial, have shown that radium-223 plus best standard of care (BSoC) prolongs overall survival (OS), reduces levels of the bone formation marker alkaline phosphatase (ALP), delays the onset of SSEs and is well tolerated compared with placebo plus BSoC [10–12]. A phase I study of radium-223 in Japanese patients with mCRPC has shown consistent pharmacokinetic parameters and dosimetry, as well as similar tolerability and biomarker responses [13, 14]. The aim of this phase II study was to further investigate the efficacy and safety of radium-223 in Japanese patients with symptomatic CRPC and bone metastases.

## Patients and methods

This phase II study (NCT01929655) was a multicenter, single-arm, open-label trial. The study protocol was approved by each study site’s independent ethics committee or institutional review board, and the study was conducted in accordance with the ethical principles of the Declaration of Helsinki and the International Conference on Harmonization guideline E6: Good Clinical Practice. Written informed consent was obtained from all patients.

### Selection of patients

Eligibility criteria were those of the ALSYMPCA study [11]. The study population included Japanese patients with progressive, symptomatic CRPC, with ≥2 bone metastases, and no known visceral metastases. ‘Symptomatic’ was defined as regular use of analgesic medication for cancer-related bone pain (≥level 1; World Health Organization ladder for cancer pain) or treatment history with external beam radiotherapy (EBRT) for bone pain within 12 weeks before the first dose of radium-223. Patients were allowed to receive concurrent BSoC, defined as the routine standard of care at each center (e.g., local EBRT, corticosteroids, first-generation antiandrogens, estrogens, ketoconazole, bisphosphonates, and denosumab), and had a history of, were ineligible for, or had refused docetaxel. Abiraterone and enzalutamide were not included in BSoC. Combination with chemotherapy agents was not allowed.

### Study design

All patients received intravenous administration of radium-223 at a dose of 55 kBq/kg on day 1 of each 4-week cycle for six cycles. Efficacy and safety assessments were also performed on day 1 of each cycle. Patients received the next dose provided they did not show critical toxicity. Any hematological or non-hematological toxicities were required to improve to grade 2 or lower prior to administration of the next dose. Patients entered the active follow-up period for up to 12 weeks after the end of treatment (EOT), and were followed up for survival for up to 36 months after administration of the first dose.

### Efficacy

The primary endpoint was percent change in total ALP from baseline at 12 weeks. Secondary endpoints included percent change in total ALP from baseline to EOT, total ALP response rate (percent of patients with ≥30 and ≥50% reduction from baseline) at 12 weeks and EOT, and OS. Other efficacy endpoints included percent change in prostate-specific antigen (PSA) from baseline at 12 weeks and EOT, PSA response rate (as in ALP), and time to first SSE (the first use of EBRT to relieve skeletal symptoms, new symptomatic pathological bone fractures, spinal cord compression or tumor-related orthopedic surgical intervention).

### Safety

Adverse events (AEs) were reported according to National Cancer Institute Common Terminology Criteria for Adverse Events version 4.03, and assessed for causal relationship with radium-223. Treatment-emergent adverse events (TEAEs) were defined as adverse events occurring or worsening after the first administration and within 30 days after the last dose of radium-223.

### Statistical analyses

The ALSYMPCA study reported a mean 32% reduction in total ALP from baseline at 12 weeks for radium-223 compared with a 37% increase for placebo, with a standard deviation of approximately 40% [12]. The midpoint between 37% increase and 32% decrease (a 2.5% increase) was rounded down to zero to be conservative and set as the threshold to indicate consistency in efficacy. If the upper limit of the 95% confidence interval (CI) for percent change in total ALP was less than zero, the consistency for total ALP between results of the ALSYMPCA study and the current study was considered met. Assuming the true percent change in total ALP in the present study is –20%, a statistical power of 90% will be obtained with 43 patients.

Statistical analyses for the study were performed using Statistical Analysis System version 9.2. All patients who received at least one dose of study medication were included in the safety analysis and those who also had at least one total ALP data point were included in the efficacy analysis. For the primary endpoint analysis, missing ALP values at 12 weeks were imputed by the last observation carried forward (LOCF) method.

The total ALP and PSA values, changes from baseline, and percent changes from baseline were summarized by visit. Kaplan–Meier estimates were plotted for time-to-event data. Median follow-up for OS was estimated by the reverse Kaplan–Meier method [15]. Post-hoc analyses on the effect of prior treatment with docetaxel on efficacy and safety were also performed.

## Results

### Patient disposition and baseline characteristics

The study was conducted at 17 centers in Japan and screened 67 patients between September 2013 and May 2014, 49 of whom received at least one administration of radium-223. The data cut-off date for this analysis was 2 February 2015 (supplementary Fig. S1).

The median age of patients was 74 years and the median Gleason score was 9. Most patients had an Eastern Cooperative Oncology Group (ECOG) performance status of 0 or 1 (69 and 27%) and an extent of disease (EOD) of 2 or 3 (39 and 53% of patients, respectively); median PSA and ALP values were 73 μg/L and 316 U/L, respectively. More than half of the patients (55%) had previously received docetaxel, and thirty-one patients (63%) received concomitant treatment with bone-modifying agents (bisphosphonates 29%; denosumab 35%). Patient characteristics are shown in supplementary Table S1.

### Exposure

The median number of radium-223 administrations was 6 (range 1–6). Twenty-eight patients (57%) completed all six administrations of radium-223. The primary reason for discontinuation (*n* = 21) was disease progression (clinical, *n* = 12, 57%; radiological, *n* = 4, 19%). Two patients (4%) discontinued study treatment due to AEs not associated with clinical disease progression (supplementary Fig. S1).

### Efficacy

#### ALP dynamics and response

**Fig. 1:**
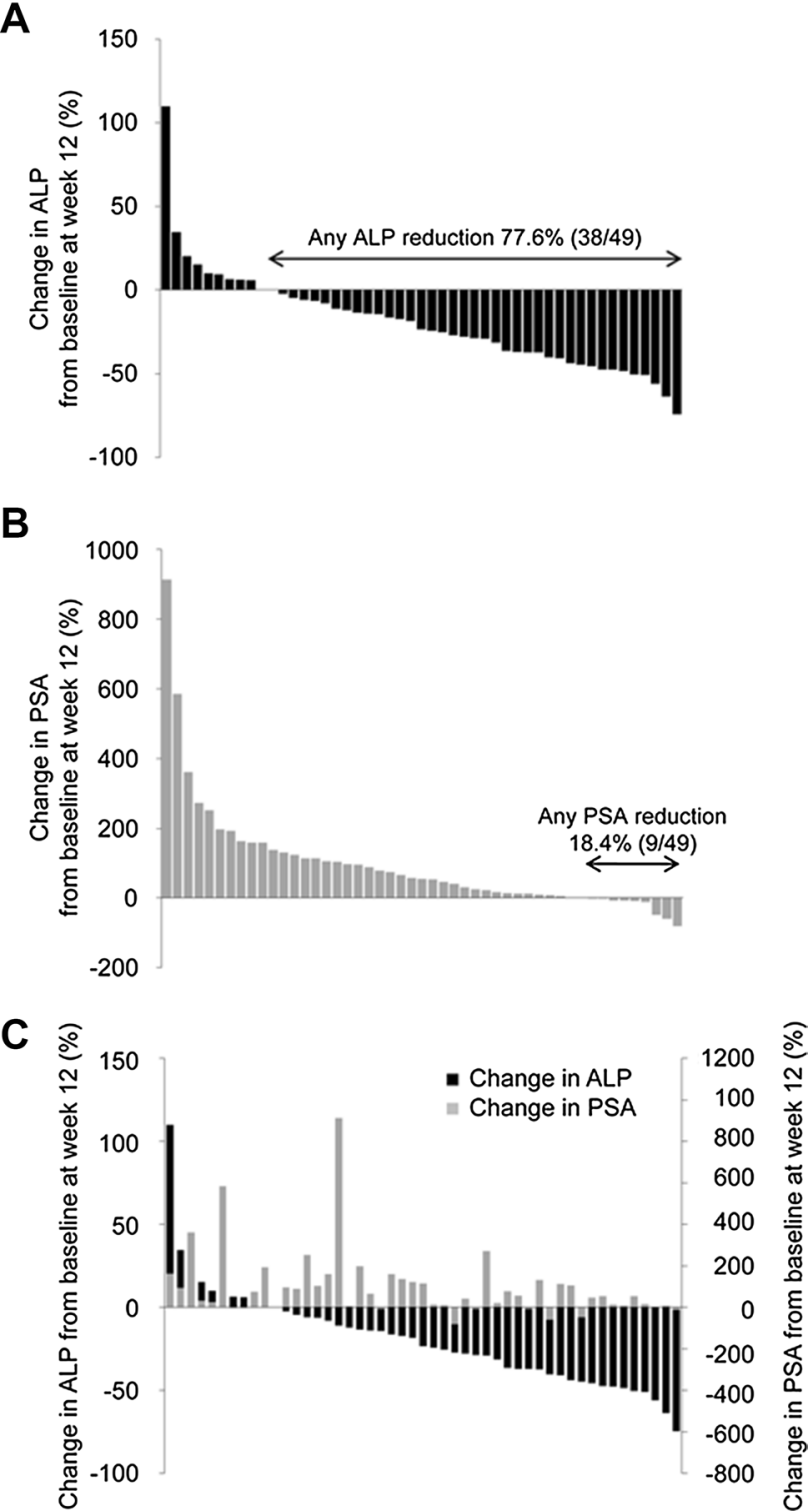
Waterfall plots showing percent change in **a** alkaline phosphatase (ALP) levels; **b** prostate-specific antigen (PSA) levels; and **c** both ALP and PSA levels for individual patients

The mean percent change in total ALP from baseline at week 12 was −19.3% (95% CI −28.0, −10.7) [Table 1] and the upper limit of 95% CI was below 0. At EOT, the mean percent change was −1.9% (95% CI −19.7%, +15.8%). Total ALP values (without LOCF) were below baseline at all time-points, with the greatest mean percent change at week 12 (mean ± SD, −20.8 ± 30.4%) [supplementary Fig. S2]. The majority of patients (78%) experienced any degree of ALP decrease at week 12 (Fig. 1a). Confirmed ALP response rates of ≥30% and ≥50% reduction from baseline were 31% and 10%, respectively, at week 12 (Table 1).

**Table 1:** Change from the baseline and response rates of ALP and PSA

	ALP		PSA	
	At 12 weeks	At EOT	At 12 weeks	At EOT
Change from baseline, % (*N* = 49)				
Mean ± SD	−19.3 ± 30.1	−1.9 ± 61.9	97.4 ± 164.9	280.5 ± 500.8
Median	−23.5	−13.8	54.4	86.1
Min	−75.0	−65.0	–81.0	–99.0
Max	110.0	262.0	914.0	2403.0
95% CI	−28.0, −10.7	−19.7, 15.8	50.1, 144.8	136.7, 424.4
Response rate, *n* (%)				
≥30% reduction				
Unconfirmed	18 (37)	17 (35)	3 (6)	4 (8)
Confirmed^a^	15 (31)	11 (22)	3 (6)	4 (8)
≥50% reduction				
Unconfirmed	5 (10)	7 (14)	2 (4)	3 (6)
Confirmed^a^	5 (10)	4 (8)	2 (4)	3 (6)

Missing values at week 12 or EOT were imputed by carrying forward the last observation including baseline (LOCF)

*ALP* alkaline phosphatase, *CI* confidence intervals, *EOT* end of treatment, *LOCF* last observation carried forward, *PSA* prostate-specific antigen, *SD* standard deviation

^a^ Confirmed by a second value approximately ≥4 weeks later

#### PSA dynamics and response

The mean percent change in PSA from baseline was +97.4% at week 12 and +280.5% at EOT, and median change in PSA from baseline was +54.4% at week 12 and +86.1% at EOT (Table 1). Confirmed PSA response rates of ≥30% and ≥50% reduction from baseline were 6% and 4%, respectively, at week 12. A PSA reduction at week 12 was observed in only nine patients (18%, Fig. 1b). The correlation between changes in ALP and PSA levels was not obvious in individual patients (Fig. 1c).

#### Overall survival and symptomatic skeletal events

**Fig. 2:**
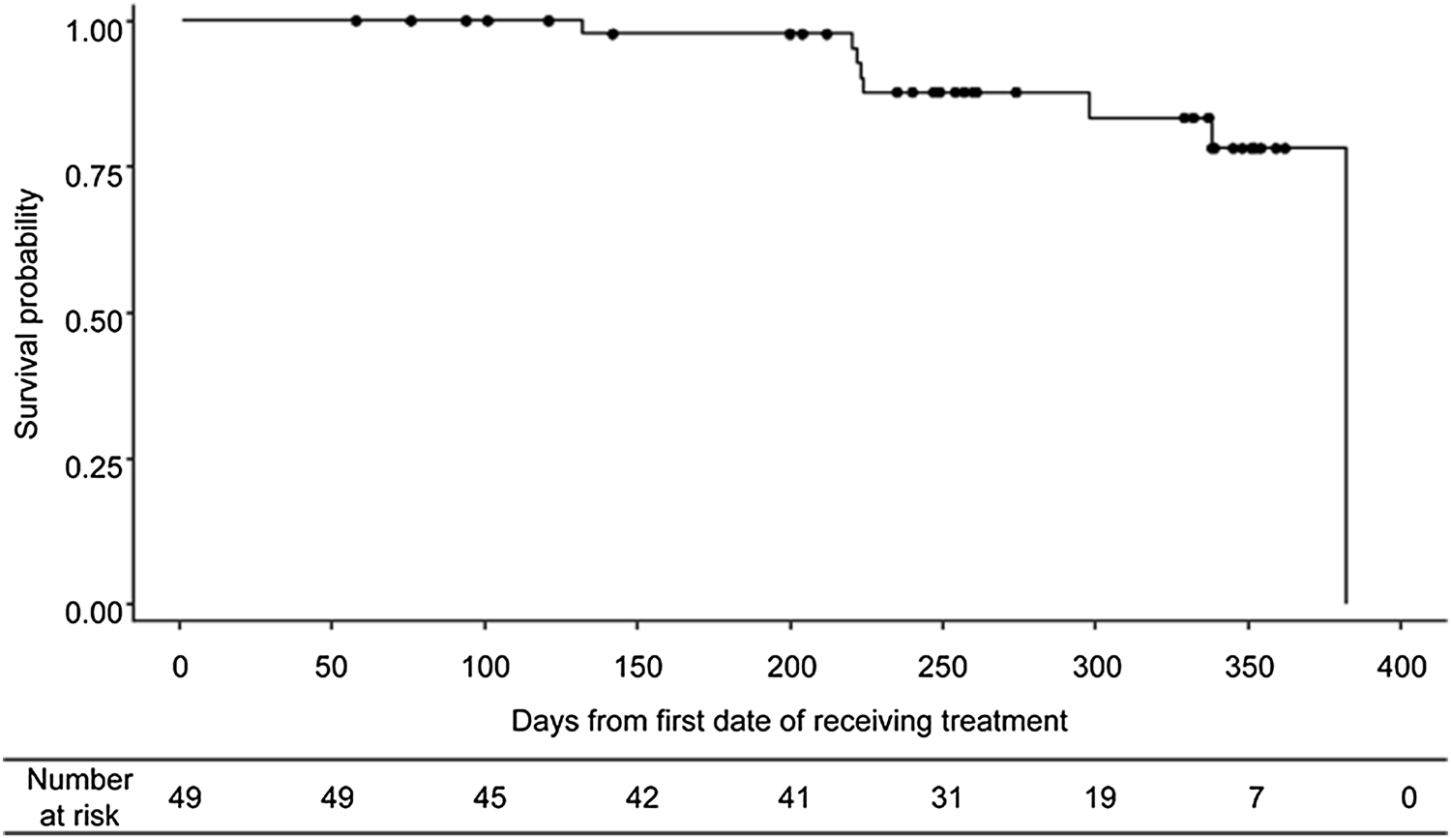
Kaplan–Meier curve for overall survival

Estimated median follow-up for OS was 8.5 months (95%CI, 8.3, 11.1). The 6-month and 1-year OS rate was 98 and 78%, respectively. The median OS of 12.5 months (Fig. 2) was based on the vertical drop on the Kaplan–Meier curve, caused by an event in a single patient with the longest observation period. The median time to first SSE was not reached (supplementary Fig. S3) and the SSE-free rate at the end of active follow-up (day 273) was 89%.

### Safety

Of the 49 patients, 44 (89.8%) experienced TEAEs and 27 (55%) experienced drug-related TEAEs (Table 2). The most common TEAEs were anemia (33%), decreased lymphocyte count (31%), anorexia (27%), nausea (25%), and bone pain (22%). Grade 3 or 4 TEAEs observed in ≥10% of patients included decreased lymphocyte count (14%), anemia (14%), anorexia (10%), and bone pain (10%). Only one patient (2%) experienced a grade 4 TEAE (decreased lymphocyte count), which was considered to be related to radium-223 by the investigator.

**Table 2:** Treatment-emergent adverse events and treatment-emergent serious adverse events by Common Terminology Criteria for Adverse Events reported in ≥5% of patients (*N* = 49)

TEAEs, *n* (%)	All grade	Grade ≥ 3^a^
Any	44 (90)	19 (39)
Hematological AE		
Anemia	16 (33)	7 (14)
Lymphocyte count decreased	15 (31)	7 (14)
Platelet count decreased	8 (16)	1 (2)
White blood cell decreased	5 (10)	0
Neutrophil count decreased	3 (6)	0
Non-hematological AE		
Anorexia	13 (27)	5 (10)
Nausea	12 (24)	0
Bone pain	11 (22)	5 (10)
Constipation	8 (16)	0
Weight loss	8 (16)	0
Dental caries	4 (8)	2 (4)
Diarrhea	7 (14)	0
Malaise	6 (12)	0
Vomiting	5 (10)	0
Dental caries	4 (8)	2 (4)
Hypophosphatemia	3 (6)	2 (4)
Aspartate aminotransferase increased	3 (6)	1 (2)
Edema limbs	3 (6)	0
Renal and urinary disorders—other^b^	3 (6)	0
Skin infection	3 (6)	0
Upper respiratory infection	3 (6)	0

Treatment-emergent SAEs, *n* (%)	All	Drug-related
Any	12 (24)	3 (6)
Bone pain	5 (10)	1 (2)
Anemia	1 (2)	1 (2)
Blood and lymphatic system disorders—other^c^	1 (2)	1 (2)
Anorexia	1 (2)	0
Gum infection	1 (2)	0
General disorders and administration site conditions—other^d^	1 (2)	0
Platelet count decreased	1 (2)	0
Pneumonia	1 (2)	0
Tumor pain	1 (2)	0

*TEAEs* treatment-emergent adverse events, *SAEs* serious adverse events

^a^ One case of grade 4 decreased lymphocyte count and remaining grade 3 AEs

^b^ Increased blood urea nitrogen, renal disorders, and deterioration of renal dysfunction

^c^ Pancytopenia

^d^ Aggravation of prostate cancer

TEAEs leading to permanent discontinuation were reported in three patients (6%); two due to grade 3 drug-related anemia, and one due to disease progression which was irregularly captured as AE by the investigator. There were no deaths reported during study treatment or within 30 days after the last administration of study treatment.

### Efficacy and safety according to prior docetaxel use

Of 49 patients included in the study, 27 had received docetaxel treatment before enrollment (supplementary Table S2). Baseline characteristics such as age, body weight, and Gleason scores were similar between patients with or without prior docetaxel, while ECOG performance status was slightly worse and EOD was greater in patients who had not received prior docetaxel.

A reduction in total ALP from baseline was observed at week 12 in 74% (20/27) of patients who had received prior docetaxel (supplementary Fig. S4a), and in 82% (18/22) of patients without prior docetaxel (Fig. S4b).

Patients with a history of prior docetaxel had a numerically higher incidence of hematological AEs than patient without, including anemia (41 vs 23%) and decreased lymphocyte and neutrophil counts (41 vs 18% and 11 vs 0%, respectively) (Table 3).

**Table 3:** Incidence of treatment-emergent adverse events according to prior use of docetaxel

	With prior docetaxel (*N* = 27)		Without prior docetaxel (*N* = 22)	
	All grades	Grade ≥ 3	All grades	Grade ≥ 3
TEAEs, *n* (%)				
Any	26 (96)	14 (52)	18 (82)	5 (23)
Leading to drug discontinuation	3 (11)^a^	3 (11)^a^	0	0
Leading to drug interruption	1 (4)	1 (4)	0	0
Hematological TEAEs, *n* (%)				
Anemia	11 (41)	6 (22)	5 (23)	1 (5)
Lymphocyte count decreased	11 (41)	5 (19)^c^	4 (18)	2 (9)
Platelet count decreased	5 (19)	1 (4)	3 (14)	0
White blood cell decreased	3 (11)	0	2 (9)	0
Neutrophil count decreased	3 (11)	0	0	0
Blood and lymphatic system disorders—other^b^	1 (4)	1 (4)	0	0
Treatment-emergent SAEs, *n* (%)				
Any	9 (33)	6 (22)	3 (14)	1 (5)

*SAEs* serious adverse events, *TEAEs* treatment-emergent adverse events

^a^Including one case of “aggravation of prostate cancer” reported as adverse event

^b^Pancytopenia

^c^Including one grade 4 AE

## Discussion

Elevated levels of ALP, a marker of bone formation, have been associated with poor prognosis in patients with mCRPC [16–18] and an increased risk of SRE [19]. The present study showed that treatment with radium-223 caused a reduction of total ALP levels in Japanese patients, consistent with the findings from the ALSYMPCA study. Taken together with favorable OS rates (98 and 78% at 6 months and 1 year, respectively, compared with ~85% and ~60% in ALSYMPCA [11]), these results suggest that the survival benefit observed with radium-223 in the ALSYMPCA study may also apply to Japanese patients.

Similar reductions of serum ALP levels from baseline were observed in patients with or without prior docetaxel treatment. This is in line with the ALSYMPCA study, where the ALP response rate (≥30% reduction) was similar regardless of prior docetaxel treatment [20].

Our results have also shown that treatment with radium-223 was generally well tolerated. AEs of grade ≥3 included anemia, decreased lymphocyte count, and bone pain, similar to the AEs reported in the ALSYMPCA study [11]. Because myelosuppression is one of the major AEs of docetaxel [21], augmentation of myelotoxicity with radium-223 due to prior treatment with docetaxel was a potential concern. Indeed, prior docetaxel was identified as one of the risk factor for Grade 2–4 thrombocytopenia in the ALSYMPCA study [22]. In the present study, radium-223 was well tolerated in both naïve and docetaxel-treated patients, despite a relatively higher number of hematological AEs reported in docetaxel-treated patients.

As the limitation of this study, OS and SSE data are not fully mature due to the relatively short follow-up period, and the analysis by prior docetaxel use is not conclusive due to its post hoc nature. However, results from this phase II trial, including the positive ALP response and tolerability, suggest that radium-223 is an appropriate treatment option for Japanese patients with CRPC and bone metastasis, regardless of docetaxel treatment history.

In conclusion, the reduction from baseline in total ALP at 12 weeks seen in this phase II study is consistent with results from the ALSYMPCA study. Overall, radium-223 was well tolerated in Japanese patients with CRPC and bone metastases.

## Electronic supplementary material

Below is the link to the electronic supplementary material.

Supplementary material 1 (TIFF 88 kb)

Supplementary material 2 (TIFF 73 kb)

Supplementary material 3 (TIFF 45 kb)

Supplementary material 4 (TIFF 85 kb)

Supplementary material 5 (DOCX 45 kb)

Supplementary material 6 (DOCX 45 kb)
