# Supplementary figures and images for: Phase II study of radium-223 dichloride in Japanese patients with symptomatic castration-resistant prostate cancer
Source: Int J Clin Oncol. 2017 Aug 2;23(1):173–80. doi: 10.1007/s10147-017-1176-0 (PMC5809574; doi:10.1007/s10147-017-1176-0)

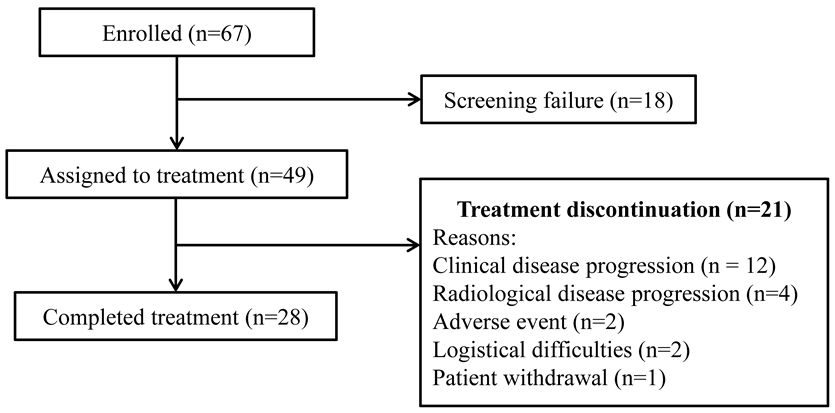

Supplement: Supplementary file 1 — Supplementary material 1 (TIFF 88 kb) [file 10147_2017_1176_MOESM1_ESM.tiff]

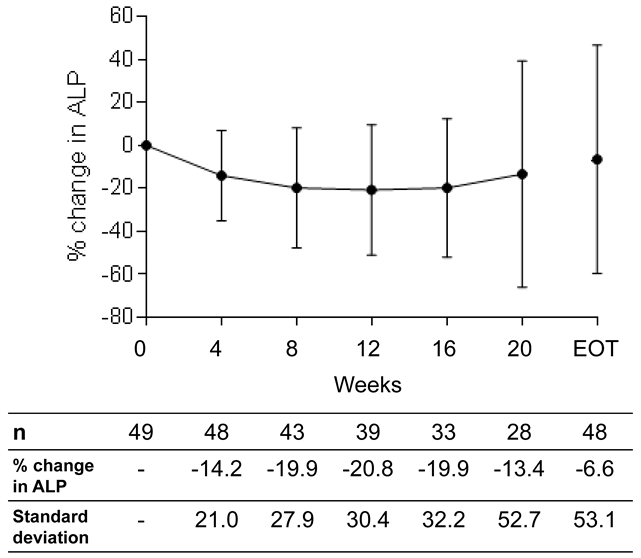

Supplement: Supplementary file 2 — Supplementary material 2 (TIFF 73 kb) [file 10147_2017_1176_MOESM2_ESM.tif]

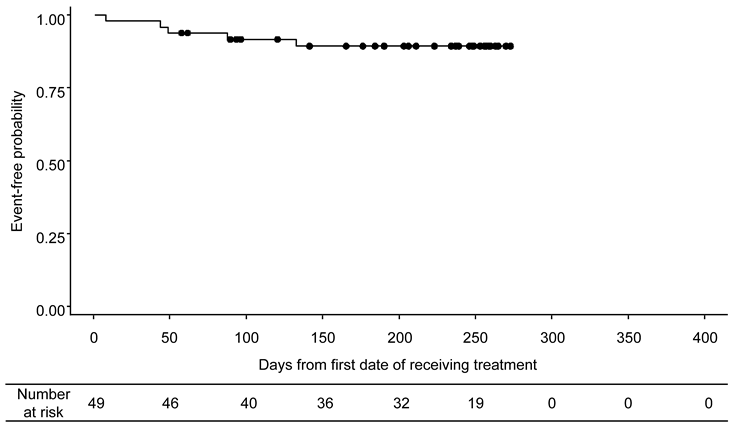

Supplement: Supplementary file 3 — Supplementary material 3 (TIFF 45 kb) [file 10147_2017_1176_MOESM3_ESM.tiff]

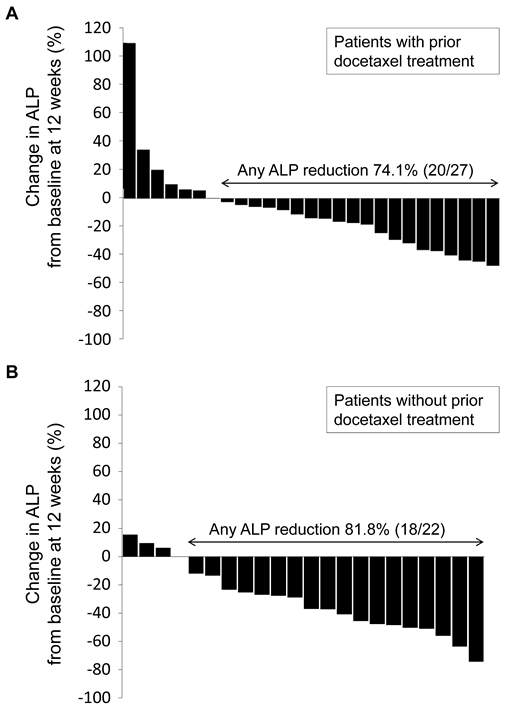

Supplement: Supplementary file 4 — Supplementary material 4 (TIFF 85 kb) [file 10147_2017_1176_MOESM4_ESM.tiff]
