# Supplementary material for: Phase II study of radium-223 dichloride in Japanese patients with symptomatic castration-resistant prostate cancer
Source: Int J Clin Oncol. 2017 Aug 2;23(1):173–80. doi: 10.1007/s10147-017-1176-0 (PMC5809574; doi:10.1007/s10147-017-1176-0)
Supplement: Supplementary file 5 — Supplementary material 5 (DOCX 45 kb) [file 10147_2017_1176_MOESM5_ESM.docx]

**Table S1.** Patient demographics and baseline characteristics.

| **Demographics and baseline characteristics** | **N=49** |
| --- | --- |
| Sex, n (%)  Male | 49 (100) |
| Race, n (%)  Asian | 49 (100) |
| Age, years  Median (range) | 74 (61−83) |
| Bodyweight, kg  Median (range) | 65 (41−85) |
| Height, cm  Median (range) | 165 (148−180) |
| BMI, kg/m^2^  Median (range) | 24.0 (16.7−30.7) |
| ECOG performance status, n (%)  0  1  2 | 34 (69)  13 (27)  2 (4) |
| Histology, n (%)  Adenocarcinoma | 49 (100) |
| TNM classification, n (%)  Stage IV | 49 (100) |
| Median Gleason score (range) | 9 (6−10) |
| EOD score, n (%)  1 (<6 metastases)  2 (6−20 metastases)  3 (>20 metastases but not superscan)  4 (superscan) | 3 (6)  19 (39)  26 (53)  1 (2) |
| Prior docetaxel, n (%)  Yes  No | 27 (55)  22 (45) |
| PSA, μg/L  Median (range) | 73 (6−2350) |
| ALP, U/L  Median (range) | 316 (82−5150) |
| Median durations (range), months  Since initial prostate cancer diagnosis  Since first progression of prostate cancer  Since diagnosis of bone metastases  Since first progression of bone metastases  Since most recent progression of bone metastases | 46.8 (7.7–167.2)  28.6 (2.1–121.1)  28.1 (2.1–166.6)  9.3 (0.1–81.9)  0.9 (0.1–60.2) |
| **Abbreviations:** ALP, alkaline phosphatase; BMI, body mass index; ECOG, Eastern Cooperative Oncology Group; EOD, extent of disease; PSA, prostate-specific antigen. | |
