# Supplementary material for: Phase II study of radium-223 dichloride in Japanese patients with symptomatic castration-resistant prostate cancer
Source: Int J Clin Oncol. 2017 Aug 2;23(1):173–80. doi: 10.1007/s10147-017-1176-0 (PMC5809574; doi:10.1007/s10147-017-1176-0)
Supplement: Supplementary file 6 — Supplementary material 6 (DOCX 45 kb) [file 10147_2017_1176_MOESM6_ESM.docx]

**Table S2** Patient and disease background with/without prior docetaxel use (n=49).

| **Baseline characteristics** | **With prior docetaxel**  **(n=27)** | **Without prior docetaxel**  **(n=22)** |
| --- | --- | --- |
| Age, years  Median (Range) | 74 (61–80) | 77 (61–83) |
| Body weight, kg  Median (Range) | 66(47–82) | 63 (41–85) |
| ECOG performance status, n (%)  0  1  2 | 22 (81)  4 (15)  1 (4) | 12 (55)  9 (41)  1 (5) |
| Gleason score  Median (Range) | 9 (6–10) | 8/9 (6–9) |
| EOD score, n (%)  1  2  3  4 | 1 (4)  13 (48)  13 (48)  0 | 2 (9)  6 (27)  13 (59)  1 (5) |
| PSA, μg/L  Median (Range) | 70 (6–2350) | 93 (9–1250) |
| ALP, U/L  Median (Range) | 264 (114–2145) | 362 (82–5150) |
| Prostate cancer, Median (Range)  Time from initial diagnosis, weeks  Time from first progression, weeks | 58.5 (20.4–167.2)  36.2 (4.2–121.1) | 29.6 (7.7–121.1)  15.2 (2.1–108.9) |
| Bone metastasis, Median (Range)  Time from initial progression, weeks  Time from last progression, weeks | 14.1 (0.5–81.9)  1.2 (0.2–60.2) | 3.1 (0.1–52.6)  0.6 (0.1–9.4) |
| ALP, alkaline phosphatase; ECOG, Eastern Cooperative Oncology Group; PSA, prostate-specific antigen. | | |
